# Assessing EM Patient Safety and Quality Improvement Milestones Using a Novel Debate Format

**DOI:** 10.5811/westjem.2015.9.27269

**Published:** 2015-11-12

**Authors:** Mira Mamtani, Kevin R. Scott, Francis J. DeRoos, Lauren W. Conlon

**Affiliations:** University of Pennsylvania Perelman School of Medicine, Department of Emergency Medicine, Philadelphia, Pennsylvania

## Abstract

Graduate medical education is increasingly focused on patient safety and quality improvement; training programs must adapt their curriculum to address these changes. We propose a novel curriculum for emergency medicine (EM) residency training programs specifically addressing patient safety, systems-based management, and practice-based performance improvement, called “EM Debates.” Following implementation of this educational curriculum, we performed a cross-sectional study to evaluate the curriculum through resident self-assessment. Additionally, a cross-sectional study to determine the ED clinical competency committee’s (CCC) ability to assess residents on specific competencies was performed. Residents were overall very positive towards the implementation of the debates. Of those participating in a debate, 71% felt that it improved their individual performance within a specific topic, and 100% of those that led a debate felt that they could propose an evidence-based approach to a specific topic. The CCC found that it was easier to assess milestones in patient safety, systems-based management, and practice-based performance improvement (sub-competencies 16, 17, and 19) compared to prior to the implementation of the debates. The debates have been a helpful venue to teach EM residents about patient safety concepts, identifying medical errors, and process improvement.

## BACKGROUND

Educating the next generation of resident physicians includes not only specialty-specific content but also patient-centered training focusing on value-based, high-quality care.^1^ In early 2000, the Institute of Medicine released their report, “To Err is Human,” highlighting the prevalence of medical error and suboptimal care.^2^ Since then, patient safety and quality improvement has become a more visible topic to hospital management, the public, and medical educators. In 2012 the Emergency Medicine (EM) Milestone Project was created by the American Board of EM (ABEM) and the Accreditation Council for Graduate Medical Education (ACGME). The EM Milestone Project is a framework of assessing competencies within several domains of EM skill sets, including sub-competencies in patient safety, systems-based management, and practice-based performance improvement (sub-competencies 16, 17, and 19).^3^ The ACGME further emphasized a learning environment focusing on patient safety and quality improvement by implementing the Next Accreditation System (NAS) in 2013.^1^

Educating resident physicians on quality improvement (QI) and patient safety has the potential to reduce errors and improve patient outcomes. In 2007, a systematic review concluded that teaching quality improvement to clinicians improved their knowledge and confidence.^4^ Several studies have shown that participation in a QI curriculum resulted in significant improvement in processes of care.^5^–^9^ A separate study published in 2010 reviewed barriers of effectively teaching quality improvement and patient safety to learners, which emphasized achieving the appropriate balance of didactic and experiential learning, as well as scheduling the curriculum amidst preexisting rotations.^10^

The authors describe a curriculum for EM residents, “EM Debates,” which combine didactic and experiential learning during resident conference as a method to teach and assess specific sub-competencies within the EM milestones. We sought to evaluate the effectiveness of this curriculum by surveying the residents’ perception of the “EM Debates,” and to determine whether the CCC could more easily assess specific patient safety and quality improvement milestones.

### Objectives

The proposed curriculum, “EM Debates,” involves a mock clinical case that is debated by two teams: a senior-level resident and attending team debating against another senior-level resident and attending team. A moderator introduces the topic and poses questions to the debaters as well as the audience participants. The teams have 15 minutes each to present their opposing views for the diagnosis, treatment, and/or disposition of commonly encountered emergency department (ED) presentations using the best available evidence. Following this, the audience participants have 15 minutes to discuss the opposing viewpoints and to determine if a consensus can be reached.

For example, a recent “EM Debate” involved the treatment of patients with atrial fibrillation. A case of a patient with acute onset atrial fibrillation who had no comorbidities and no symptoms was presented. Articles were reviewed arguing that patients should be cardioverted in the ED and discharged home. The opposing team reviewed articles arguing that patients should be anticoagulated and placed in the hospital for delayed cardioversion. Following the discussion, the conference attendees (residents, faculty, and nurses) debated the relative merits of each side and eventually came to a consensus. The residents who presented the debate then worked with key QI faculty members within our department and the department of cardiology to create a pathway for patients with atrial fibrillation.

The objectives of this curriculum are separated for those who participate in an “EM Debate,” lead a debate, and develop a protocol following the completion of the debate. The goals for the debate leader include developing an in-depth understanding of a controversial topic, critically analyzing current literature with a faculty mentor, and creating a persuasive argument to teach the participants about managing a specific disease process. The debate leaders could be assessed on leading team reflections to improve ED performance and demonstrating evidence-based information retrieval mastery. These assessments would fall within patient safety sub-competency 16 and practice-based performance improvement sub-competency 19.

The goals for the debate participants, those residents in the audience, include describing the best available evidence or controversies surrounding a specific topic. Additionally, participants will appraise the value of the leader’s presentations and choose a management strategy they will adopt for a specific clinical question. The debate participants could be assessed on their ability to describe patient safety concepts (sub-competency 16, level 3) and identifying situations when breakdown in teamwork or communication may contribute to medical error (sub-competency 16, level 4). Additionally, the participants could be assessed on the ability to call effectively on other resources in the system (sub-competency 17, level 3), and the ability to critically appraise literature and apply evidence-based medicine (sub-competency 19, level 3).

The goals for the debate leaders who participate in protocol development following a debate include synthesizing the current literature and the feedback from the participants during the debate to propose a clinical pathway for departmental practice. By completing the pathway, the senior level resident will have addressed sub-competency 19 at level 4; specifically, they would have participated in a process improvement plan to optimize ED performance and applied performance improvement methodologies. Furthermore, they will have addressed sub-competency 17 at level 4; specifically, they would have participated in processes and logistics to improve patient flow and decrease turnaround time.

### Curricular Design

This curriculum was guided by the six-step model for medical curriculum development. The six-step approach highlights a process of general needs assessment, targeted needs assessment, goals and objectives, educational strategies, implementation, and evaluation and feedback.^11^

Implementation of the EM milestones highlighted the general needs assessment within graduate medical education on teaching patient safety and QI. Within our program we targeted controversial topics that were seen as patient safety issues. We felt our learners would benefit from active participation in evidence-based discussions regarding clinical management strategies and for our senior residents to develop protocols with department leadership. The goals and objectives outlined above directly address sub-competencies within the EM milestones. Using a debate format we were able to engage learners at various levels and promote discussion. Implementation during regular EM conference time maximized resident and faculty participation. Additionally, securing continuing medical education credit under the patient safety designation for the “EM debates” offered added incentive for faculty attendance. Evaluation and feedback of our curriculum are ongoing and will be discussed later in this manuscript.

## IMPACT/EFFECTIVENESS

The “EM Debates” were implemented two years ago at our institution. We conducted a cross-sectional study to evaluate the effectiveness of this new curriculum. The survey was distributed to the EM residents at a single site to determine their perceptions of the debates using a Likert scale. The responses were anonymous and participation was voluntary. Questions were divided into sections based on whether the respondent participated in a debate, led a debate, or were working on developing a pathway for the department after completion of a debate. The questions are linked to specific milestones. For example, one question: “After participating in a debate, I feel I have improved my individual performance on a specific topic by critically appraising scientific literature and applying evidence-based medicine,” is linked to sub-competency 16, level 4 of the EM milestones.

We also conducted a cross-sectional study to determine the clinical competency committee’s (CCC’s) ability to assess residents on specific competencies. We sent a separate survey to CCC members to assess whether it was easier to assess these competencies after the implementation of the “EM Debates.” Again, the responses were anonymous and participation was voluntary.

The institutional review board at our site granted exempted approval to this study. We created and distributed the survey using the online survey tool SurveyMonkey.^©^ Survey responses were collected and compiled. We used descriptive quantitative and qualitative statistics to assess survey responses.

## RESULTS

### Quantitative

#### Residents

The survey was sent to 42 residents; of those, 30 residents responded (71% response rate). Seventy-one percent (71%) of these residents agreed or strongly agreed that they have improved their individual performance on a specific topic by critically appraising scientific literature and applying evidence-based medicine, which linked to sub-competency 19, level 3. Sixty-eight percent (68%) agreed or strongly agreed they could describe patient safety concepts, like the “Swiss cheese” or “near miss” model, which linked to sub-competency 16, level 3.

Approximately a third of the residents have reported that they have led a debate. Of those, 100% felt that they agreed or strongly agreed that they can propose an evidence-based approach to a specific topic, and 91% agreed or strongly agreed that they have analyzed or worked on improving ED performance, correlating to sub-competency 16 (level 4) and 19 (level 4), respectively.

Of those that led a debate, approximately half responded that they have worked on a protocol for the department. As only our third-year residents have led a debate, and we have completed half of them this year, this is an accurate representation. Of these residents, 100% agree or strongly agree that they have applied performance improvement methodologies and have analyzed processes and logistics to improve patient flow and turnaround time, corresponding to sub-competency 19 (level 4) and 17 (level 4), respectively.

#### CCC

The CCC felt that it was easier to assess sub-competency 16 levels 3 and 4, sub-competency 17 levels 3 and 4, and sub-competency 19 levels 3 and 4 as compared to prior to the implementation of the debates.

### Qualitative

Respondents were overall very positive towards the implementation of the debates. A majority of the residents commented on the engaging aspect of discussing opposing management strategies of controversial topics, while others valued the detailed literature review. Some respondents, particularly junior residents, would appreciate more background information at the beginning of the debate to understand why the clinical case can have various management options. Additionally, multiple respondents felt that additional time devoted to the “EM Debates” during conference would be helpful.

## LIMITATIONS

While the evaluation of the curriculum “EM Debates” indicated that the residents enjoyed it and, in certain instances, the pathway development changed clinical practice, it does not reveal whether it changed the residents’ behavior. Future studies could be performed using direct observation during clinical practice to determine whether residents’ behavior has changed as a result of this curriculum.

Furthermore, assessment of the debate leaders and the audience participants relies on subjective data. The assessment of the residents could be more robust if faculty with expertise in the area reviewed videotapes of the “EM Debates” to characterize the strength and validity of the debate leaders’ argument and the audience participants’ involvement in the discussion. A potential method to assess participants could include a post-debate examination to determine their level of understanding and ability to critically appraise the discussed literature. This would provide the CCC with more objective data of the residents’ abilities.

This study was performed at a single site; further studies would need to be performed at additional sites to evaluate whether hospitals with different resources and cultures would find this as helpful.

## DISCUSSION

Implementation of this curriculum is directly applicable and feasible among other EM programs. While faculty involvement could be a barrier, core faculty can be used as faculty mentors for the residents.

One challenge we noticed was the process of creating a protocol for the department is predicated on agreement when debating specific topics. If agreement is not reached, then the residents will delay creating a protocol until consensus within the department can be reached. Thus, specific milestones will not be assessed for those who have led a debate but were unable to develop a protocol. For those residents, we found that assigning them to help with the creation of other protocols in which consensus was reached was a reasonable alternative. Furthermore, the success of pathway development is inherently dependent upon collegial interdepartmental relationships; inclusion of outside departments during the debates can help facilitate this.

While this is not a comprehensive quality and safety curriculum, it does include five of the eight Institute for Healthcare Improvement domains for health professional students: healthcare as a process, variation and measurement, collaboration, leading, following, and making changes, and developing new locally useful knowledge.^12^

The residents are generally very positive towards the “EM Debates,” and it has been a helpful venue in teaching the residents about patient safety concepts, identifying medical errors, and process improvement. In addition, these debates have made it easier for the CCC to assess the sub-competencies practice-based performance improvement, patient safety, and systems-based management within the EM Milestone Project.

## References

[1] Nasca TJ, Philibert I, Brigham T (2012). The next GME accreditation system rationale and benefits. N Engl J Med.

[2] Kohn LT, Corrigan J, Donaldson MS (2000). To Err Is Human: Building a Safer Health System.

[3] Accreditation Council for Graduate Medical Education (ACGME) The Emergency Medicine Milestone Project.

[4] Boonyasai RT, Windish DM, Chakraborti C (2007). Effectiveness of teaching quality improvement to clinicians: a systematic review. JAMA.

[5] Coleman MT, Nasraty S, Ostapchuk M (2003). Introducing practice-based learning and improvement ACGME core competencies into a family medicine residency curriculum. Jt Comm J Qual Saf.

[6] Holmboe ES, Prince L, Green M (2005). Teaching and improving quality of care in a primary care internal medicine residency clinic. Acad Med.

[7] Mohr JJ, Randolph GD, Laughon MM (2003). Integrating improvement competencies into residency education: A pilot project from a pediatric continuity clinic. Ambul Pediatr.

[8] Oyler J, Vinci L, Arora V (2008). Teaching internal medicine residents quality improvement techniques using the ABIMs practice improvement modules. J Gen Intern Med.

[9] Weingart SN, Tess A, Driver J (2004). Creating a quality improvement elective for medical house officers. J Gen Intern Med.

[10] Wong BM, Etchells EE, Kuper A (2010). Teaching quality improvement and patient safety to trainees: a systematic review. Acad Med.

[11] Kern DE (1998). Curriculum development for medical education: a six step approach.

[12] Ogrinc G, Headrick LA, Mutha S (2003). A framework for teaching medical students and residents about practice based learning and improvement, synthesized from a literature review. Academic Medicine.

